# Characterizing In Vivo Exposure to Alcohol Content in the Media: Media Platform, Engagement, and Associations With Attitudes

**DOI:** 10.1111/acer.70311

**Published:** 2026-06-12

**Authors:** Kristina M. Jackson, Joy Gabrielli, Suzanne M. Colby, Tyler B. Wray, Michelle L. Rogers, Tim Janssen, Erin Corcoran, Shimei Nelapati, Alex Clement, Matthew Meisel

**Affiliations:** ^1^ Rutgers Addiction Research Center, Department of Psychiatry Rutgers Robert Wood Johnson Medical School Piscataway New Jersey USA; ^2^ Department of Clinical and Health Psychology & Center for Addiction Research and Education University of Florida Gainesville Florida USA; ^3^ Center for Alcohol and Addiction Studies Brown University School of Public Health Providence Rhode Island USA; ^4^ Department of Behavioral and Social Sciences Brown University School of Public Health Providence Rhode Island USA

**Keywords:** adolescent, alcohol, attitudes, ecological, media

## Abstract

**Background:**

Alcohol‐related content in media is overwhelmingly presented positively, and exposure to this content is linked to adolescent drinking. Yet, less is known about how often adolescents encounter alcohol content via media, how exposure varies across platforms, and how adolescents typically engage with such content. We used ecological momentary assessment (EMA) methods to collect detailed, real‐time data about alcohol‐related content encountered across a variety of digital media sources.

**Methods:**

High school students recruited through social media (*N* = 302; mean age = 16.2; 63.9% female sex) completed three 21‐day EMA bursts spaced 4 months apart. Participants reported alcohol‐related content encountered via scheduled and self‐initiated smartphone surveys, yielding 55,352 total reports, including 8792 with alcohol content. We fit multilevel models to evaluate momentary associations between exposure to alcohol content and cognitive outcomes (personal attitudes, perceived peer norms, beliefs that content promotes alcohol use).

**Results:**

Nearly all participants (99.3%) reported exposure to alcohol‐related content during the monitoring period. The most common sources were Instagram (32.4% of alcohol reports), TikTok (18.1%), and YouTube (7.8%). About one‐third of exposures originated from influencers; nearly half of YouTube and Instagram exposures involved industry sources. In‐depth engagement (e.g., commenting) with content was rare, although engagement requiring minimal effort (“liking”) was endorsed for 25.6% of content reports. Encountering alcohol content on Instagram and Snapchat was associated with personal disapproval of the content; there were few associations with perceived peer norms. Engagement significantly predicted more cognitions favoring alcohol.

**Conclusions:**

Adolescents frequently encounter alcohol content across diverse platforms, often from influencers and industry. Much of the content was believed to promote alcohol use. Although behavioral engagement was limited, exposure was linked to attitudes toward content encountered on some platforms. Greater understanding of components and contexts to be targeted for just‐in‐time interventions delivered in proximity to in vivo exposures can reduce risk for early alcohol engagement.

## Graphical abstract

Media alcohol‐related content (ARC) is prevalent and associated with adolescent drinking. This study collected real‐time data about ARC exposure across multiple digital media sources. Many of the 8792 ARC exposures originated from influencers or the alcohol industry. Most content was believed to promote alcohol use, although engagement with content was rare. Personal disapproval of ARC was greatest on Instagram and Snapchat. Understanding components and contexts for interventions delivered in proximity to ARC exposure will reduce risk for early alcohol engagement.

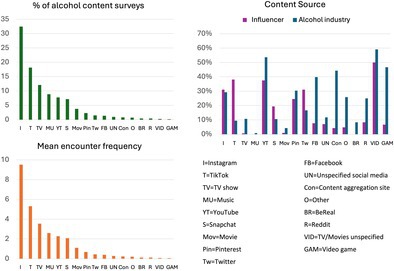

## 1 Introduction

Early to mid‐adolescence is the peak period of risk for alcohol initiation, and although adolescent alcohol use is on the decline (Rossow et al. 2020), the prevalence is still concerningly high (Miech et al. 2025). Strong and consistent associations between underage drinking and adverse outcomes have been demonstrated (Boden et al. 2020; Lees et al. 2020; World Health Organisation 2014; Yuen et al. 2020). However, despite the known impact of early alcohol use, reducing underage drinking proves challenging (Glenn et al. 2023). The alcohol research field increasingly recognizes the role of the environment in shaping underage drinking, with media being an important contextual influence. Alcohol content in the media is overwhelmingly positive in nature, with few depictions of negative consequences (Beullens and Schepers 2013; Cavazos‐Rehg et al. 2015; Clement et al. 2025; Hendriks et al. 2018; Mandzufas et al. 2024; Primack et al. 2015, Primack et al. 2008; Rutherford et al. 2023; Stern and Morr 2013). Over the past two decades, positive, predictive associations between exposure to alcohol content in the media and adolescent alcohol involvement have been consistently demonstrated (Alhabash et al. 2022; Anderson et al. 2009; Cheng et al. 2024; Curtis et al. 2018; Jackson and Bartholow 2020; Steers et al. 2025), with numerous rigorous prospective studies documenting associations that are robust to potential interpersonal and individual confounders.

### 1.1 Alcohol Content and Media Platform

Historically, research on alcohol‐related media content examined associations between alcohol‐related attitudes and behavior and entertainment media. This includes media platforms such as films (Cin et al. 2009; Hanewinkel et al. 2007; Jackson et al. 2018; Mejia et al. 2016; Sargent et al. 2006; Stoolmiller et al. 2012; Wills et al. 2009) and TV shows (Austin and Meili 1994; Engels et al. 2009; Grube 1993; Stacy et al. 2004) including through popular streaming sites (Alfayad et al. 2022; Chapoton et al. 2020; Giannakodimos et al. 2022), as well as content heard in music lyrics (Alen et al. 2024; Hardcastle et al. 2015; Primack et al. 2008, 2012) and viewed on music videos (Cranwell et al. 2015). Subsequently, research turned its focus to social media platforms, initially focusing on Facebook (Boyle et al. 2016; Moreno et al. 2016, 2012) and expanding to other platforms including YouTube, Instagram, Snapchat, Twitter, and TikTok as these new platforms emerged and evolved (Boyle et al. 2016; Litt et al. 2018; Strowger et al. 2022, 2024; Strowger and Braitman 2023; Vranken et al. 2020). Studies likewise consistently indicate that exposure to social media alcohol content is associated with alcohol use and problems, both cross‐sectionally and prospectively. Far less is known about alcohol portrayals on often‐used digital platforms such as Pinterest, Discord, Tumblr, Twitch, and BeReal, although other substance use‐related content such as hookah and waterpipe smoking and e‐cigarette use have been shown on several of these platforms (Guidry et al. 2016; Lee et al. 2017; Primack et al. 2016). Thus, although both self‐reported and objectively captured exposure to alcohol content in the media are well documented in youth, there is insufficient knowledge with respect to how frequently adolescents encounter alcohol on their personal devices over the course of the day and on which media platforms they encounter and/or engage with alcohol content.

Over the past decade, the American Academy of Pediatrics has advocated for media literacy education (e.g., teaching critical thinking skills, challenging attitudes) as a strategy for reducing adolescent substance use (Council on Communications and Media 2016; Moreno et al. 2024). Although alcohol‐focused media literacy programs demonstrate success in children (Hindmarsh et al. 2015; Park et al. 2022), there is less support for efficacy in adolescents, despite the increased drinking opportunities and saliency of alcohol for this age group and their tremendous engagement with media sources. Most substance use‐related media interventions fail to consider the context in which adolescents encounter alcohol content, that is, on personalized digital devices throughout the day. Most interventions rely on example media rather than teaching recipients to process specific media they themselves observe, which may be less personally relevant and less impactful. Prevention work is frequently limited to one or a few media platforms or may reference unspecified media or collapse measurement of exposure across numerous media platforms. Finally, current programs focus on passive use of media, ignoring the high behavioral engagement with media that is characteristic of adolescent media usage. More information about the content, platforms, and engagement thus is critical for advancing adolescent media intervention programs.

### 1.2 Response to Alcohol Content

#### 1.2.1 Behavioral Engagement With Alcohol Content

Whereas simply being exposed repeatedly to alcohol content may alter subsequent alcohol‐related beliefs and behavior (“mere exposure effect”; Alhabash et al. 2022; Zajonc 1968), engaging with alcohol content such as liking, commenting, or sharing may magnify its influence (Kurten et al. 2022; McClure et al. 2013; Steers et al. 2024). Active engagement with social media content conveys positive social validation and provides peer reinforcement for such content (Moreno and Whitehill 2014). A meta‐analysis found moderate effect sizes between alcohol‐related social media engagement and young adult alcohol use and problems (Curtis et al. 2018). Although young adults frequently post and engage with digital alcohol content, younger adolescents with less direct experience with alcohol may be reluctant to engage with risky content such as underage alcohol use. Relative to college students/young adults, far fewer adolescents post alcohol content (Meisel et al. 2022; Ward et al. 2022), and they may likewise be less likely to publicly comment or share encountered content. Self‐censoring behaviors are done in anticipation of the negative consequences of online behaviors (Fiesler et al. 2024; Geusens and Vranken 2021). Recent qualitative work indicated that adolescents elect not to react to substance use, even via forms of engagement requiring minimal effort such as “liking,” as a means to indicate that they do not support or want to be associated with the content (Corcoran et al. 2024). Adolescents also may be reluctant to share social media content generated from their friends as it may produce negative consequences or draw attention to illegal behaviors such as underage drinking. Less is known, though, about how youth behaviorally engage with or promote alcohol content in more private digital settings (e.g., Snapchat, through fake/spam accounts). Snapchat's platform features time‐limited content that can disappear and facilitates posting of negative alcohol consequences, including incriminating photos of alcohol misuse (Boyle et al. 2017) and that may encourage more accurate representations of alcohol's effects.

#### 1.2.2 Cognitive Reactions to Alcohol Content

Studies have found that exposure to alcohol content is associated with personal attitudes (Geusens and Beullens 2018; Roberson et al. 2018) and normative perceptions (Brown et al. 2016; de Graaf 2013; Meisel et al. 2022; Vanherle et al. 2024) about alcohol content, with mediational studies indicating that attitudes and norms are a conduit to alcohol use itself (Beullens and Vandenbosch 2016; Geusens and Beullens 2018; Nesi et al. 2017; Osberg et al. 2012; Vranken et al. 2020). This robust set of findings is in line with the reasoned action approach (RAA) (Conner et al. 2017; McEachan et al. 2016) which proposes that intention and behavior follow an individual's attitudes and perceived peer norms about that behavior (here, perceiving that a friend or peer approves of and likes the content).

In addition, although the majority of media representations of alcohol use portray alcohol in a positive light, content related to the negative effects of alcohol (e.g., vomiting, experiencing hangover symptoms, making poor decisions) and prevention messages, references to abstinence, sobriety, or avoiding drinking are also evident in content by media users and alcohol prevention or policy groups (Moreno et al. 2021). Thus, as a final objective, the present study explores cognitive responses to alcohol content, including the belief that the alcohol content is perceived as promoting (vs. discouraging) alcohol, personal attitudes, and perceived norms, as indexed by self‐reported self and others' approval and liking of the content.

### 1.3 Overview

The present study addresses a gap in the field by presenting data from an in‐depth ecological study on adolescent exposure to alcohol content in the media. The study addressed two broad aims: (1) To determine the frequency with which adolescents encounter alcohol content in digital media over the course of the day and on which specific media platforms' alcohol content is encountered. In addition, to contextualize our findings, we provide data on the participants' general use and engagement with various media platforms. (2) To examine in vivo reactions upon encountering content with respect to both behavioral engagement (e.g., likes, comments) and cognitive reactions to the content, including personal attitudes, perceived peer injunctive norms, and beliefs that the content promotes alcohol use. We also looked at the overlap among the reactions (the association between behavioral engagement and cognitive reactions). This is the first study to quantify and characterize adolescents' real world, in vivo exposure, and reactions to alcohol content across the full range of media platforms.

## 2 Materials and Methods

### 2.1 Participants

High schoolers were recruited nationwide to the Team300 Study through paid advertisements on social media (specifically, Instagram/Facebook) and through TikTok posts by research team members (see Jackson et al. 2025 for further details about recruitment procedures). Inclusion criteria were aged 15–18 years; enrolled in 9th–12th grade; owned a smartphone (iPhone or Android); and lived in the United States (verified via phone call). We only included youth who reported ever having consumed (at least) one drink of alcohol or having at least one close friend who drank alcohol, to ensure some degree of familiarity with alcohol. Ultimately, 302 participants were enrolled in four cohorts, 3 months apart (C1 = 56 participants; C2 = 101 participants; C3 = 103 participants; C4 = 42 participants). Enrolling participants in cohorts was intended to enhance the feasibility of recruitment for research staff and to permit examination of seasonality, given potential seasonal differences in response rates, alcohol use, and access to media and electronic devices (Carpenter 2003; Cho et al. 2001; Clapp et al. 2008). Recruitment materials evolved slightly across the four cohorts to maintain balance across age and sex (Gabrielli et al. 2020). In the final sample, 63.9% (193/302) of participants reported being female; gender distribution was reported as follows: 50.7% girl (cis‐ or trans‐), 31.5% boy (cis‐ or trans), and 18.5% nonbinary, gender nonconfirming, or other. Mean age was 16.21 years (SD = 0.77) and 1.3% were in 9th grade, 17.2% were in 10th grade, 44.0% were in 11th grade, and 37.4% were in 12th grade. The sample exhibited racial and ethnic heterogeneity, with 52.0% White, 25.8% Asian, 21.5% Black, 5.0% other, 3.6% American Indian (multiple response options permitted); 25.8% reported being Hispanic/Latinx.

The overall sample was relatively alcohol‐naïve, with 14.2% having no direct experience with alcohol. About half (52.0%) reported ever consuming a full drink; 28.1% reported ever consuming at least a sip of alcohol but not a full drink (excluding sipping as part of a religious occasion), and 5.3% reported sipping alcohol during a religious occasion only. Only 29.5% reported ever having gotten drunk. Among those who had ever consumed alcohol, 91.8% had consumed it within the past 12 months, 76.6% in the past 4 months, and 48.1% in the past 30 days. For those who consumed alcohol in the past 12 months, the majority drank either one to three times (42.8%) or 4–11 times (29.0%).

### 2.2 Procedure

#### 2.2.1 Study Design

The study combined a longitudinal panel design with experience sampling methods and consisted of three 21‐day bursts of daily EMA assessments separated by 4‐month intervals, with each interval preceded by longer surveys. A full description of the study protocol is provided in Jackson et al. (2025).

#### 2.2.2 Participant Orientation/Baseline Assessment

Zoom group‐orientation sessions were run in groups ranging from 2 to 10 (median = 4) participants. Orientation sessions covered general study elements, participation timelines, and compensation structure. The overall goals were to promote study engagement and teach participants: (1) examples of references to alcohol that might be encountered in day‐to‐day use of media, (2) the different types of reports, (3) how to identify each type of survey and corresponding submission details, (4) how to submit a self‐initiated report about different types of media content they noticed, and (5) how to de‐identify images and upload them to the app. Research assistants assisted participants in real time in downloading and logging into the EMA app (MetricWire) with their study‐assigned emails and adjusting phone settings and permissions. Youth were instructed to keep their device with them except while at school and sleeping and to monitor that notifications were being received. They were informed that researchers were not taking any information from their phone, putting anything on their phone or trying to change their behavior. We emphasized that participants should not deviate from their normal activities to find alcohol content and clarified that compensation was contingent on submitting a complete report rather than on amount of alcohol content reported. Following attendance at the orientation session, participants were emailed a link to a Qualtrics baseline survey to be completed prior to the first day of EMA data collection.

#### 2.2.3 Instructions for Reporting Alcohol Content

During orientation, participants were instructed on how to report each instance of media alcohol content by initiating a survey that was continuously available to them on the study app's dashboard or by reporting it in a scheduled media report. Participants were asked whether they encountered alcohol content on one or multiple instances; for multiple instances, they were instructed to think back to the most recent instance of alcohol content when completing the items. For movies, shows, and videos, participants were told to report on the most noticeable/important scene or example of alcohol content if there were multiple references to alcohol.

#### 2.2.4 EMA Protocol

Participants completed two types of EMA reports: scheduled media reports and self‐initiated reports. They received prompts to complete scheduled reports during four time blocks each day: 5 am to 2 pm (morning), 2–5 pm (afternoon), 5–8 pm (evening), and 8–11 pm (night), at random times within the respective time block. Participants had the option to complete a separate Bedtime report that could replace the random nightly scheduled report if needed (e.g., if the participant desired to go to sleep prior to receiving the nightly report notification or if they missed the nightly report notification but still wanted to submit a survey). The bedtime report was continuously available between 8 pm and midnight.

Scheduled media reports assessed whether participants had come across alcohol content since their last submitted report. Self‐initiated media reports allowed youth to record exposure to media alcohol content in vivo by initiating an assessment whenever they encountered an alcohol reference in the media. If participants encountered more than one exposure in a time block, their first self‐initiated media report fully characterized all content including cognitions and context; subsequent self‐initiated reports were abbreviated to reduce participant burden when submitting multiple exposures. All reports assessed format/medium, source, and whether the participant engaged with the content. Participants also were asked to provide a screenshot, photo, video, or text description of the content and to provide the link to the video if relevant (e.g., YouTube).

### 2.3 Measures

#### 2.3.1 Baseline Surveys

Age, grade, sex, gender, racial group, and ethnic group were reported at enrollment. Participants provided information on device access, use, data plan, and engagement. They reported number of minutes spent on a given platform on a typical weekday for the following platforms: Snapchat, Instagram, TikTok, YouTube, Facebook, BeReal, TV shows, movies, playing video games, and direct messaging. Ordinal response options of None, 30 min or less, 1, 2, and 3+ h were converted to number of minutes (0, 30, 60, 120, and 180) for the purpose of analysis. Participants were instructed to select None if they did not typically use a given platform. From these response options, we also computed the proportion of participants who reported spending an hour or more on each media platform. Where relevant, participants indicated whether they had an account for a given media platform and the number of accounts if more than one. General engagement with social media posts (e.g., “likes,” emoji reactions) was assessed, ranging from never to frequently. Finally, participants responded to a series of questions about their alcohol use, including lifetime, past 12‐month, past 4‐month, and past 30‐day use.

#### 2.3.2 EMA Measures

For all reports of media alcohol content, the survey assessed the format/medium: Snapchat, Instagram, TikTok, Facebook, YouTube, movie, TV show, music (e.g., radio, Spotify), and BeReal (platform added mid‐study). A final “other” write‐in option was coded independently by two coders after data collection commenced, yielding the additional codes of Pinterest, Reddit, Twitter, video games, other social media, non‐social media/content aggregation sites (e.g., webtoon), a combined TV/movies category, an unspecified social media category, and “other.” Open‐ended responses to the “other” category that included print media (e.g., billboard; train station ad; poster at a restaurant) or were not media platforms at all (restaurant with drinks everywhere; I walked past a bar) (*n* = 84) were not included.

For social media platforms, we assessed who posted or shared the content, with response options including sponsored ad/commercial that appeared; company/brand I follow; influencer/celebrity I follow; and friend, family member, or someone I know. We also asked respondents to report who created/made the original content. If the original content was not made by the same source, we probed who made it, using the same response options as above. Based on these items, we coded whether the content was made, posted, or shared by an influencer. Additionally, we asked whether the alcohol content was part of the movie, TV show, or YouTube video versus as part of a sponsored ad/commercial that appeared before/during the video. If sponsored ad/commercial that appeared or company/brand I follow was endorsed, or if the content was part of a sponsored ad/commercial that appeared before/during the video, we scored the content as made, posted, or shared by the alcohol industry.

For submitted social media content, participant behavioral engagement was assessed by asking them whether they engaged in any of the following: liked; other reaction (e.g., emoji); commented; shared/posted publicly; shared/posted privately; saved it or added it to a list; followed user/account or related group; blocked or deleted the user/account; clicked on an affiliated link; discussed with someone else, in person; and “nothing (i kept scrolling).” Participants reported on their own personal attitudes about the alcohol content following the exposure across two domains: (1) disapproval/approval of content and (2) disliking/liking content. Participants also reported normative perceptions about teens their age using the same two items. Each item had three response options anchored by the relevant dimension. For example, for the first item, the item “My personal feeling about this content is ___” and “The feeling most teens my age would have about this content is ___” were anchored by disapproval (1) and approval (3). A mean was calculated across the two items for personal attitudes and perceived peer norms. We also asked whether the participant thought the alcohol content discourages versus promotes alcohol/drinking across three referent groups: themselves, teens their age, and the creators of the content, with three response options anchored by discourages (1) and promotes (3). A mean was calculated across these three items (α = 0.87). A multilevel confirmatory factor analysis (CFA) for these three factors (personal attitudes, perceived peer norms, and the belief that content promotes drinking) had good fit (RMSEA = 0.03, CFI = 0.92, SRMR = 0.03), with standardized factor loadings for all seven indicators exceeding 0.70.

### 2.4 Analytic Strategy

Descriptive analyses were conducted to characterize exposure to media alcohol content (Aim 1) and behavioral engagement with the content (Aim 2). The prevalence of general media usage was also calculated. To examine associations between alcohol content and cognitive outcomes (Aim 2), a series of two‐level multilevel models were conducted with survey nested within person (Hayes 2006; Raudenbush and Bryk 2002). The three cognitive outcomes were personal attitudes about the alcohol content, perceived peer norms about the content, and beliefs about whether the content promotes alcohol use. A fourth analysis examined associations between behavioral engagement with the content and the three cognitive outcomes. Given that the three outcome variables were roughly normally distributed (skew < 1 and kurtosis < 2), we used a Gaussian distribution with an unstructured (general covariance) matrix and a random intercept and slope. For the sake of consistency across models, random slopes that were non‐significant were retained unless the model failed to converge in which case it was removed (as indicated in a table note).

Person‐mean centering was used. To isolate the within‐person effect of media platform, we included the person‐level aggregate (proportion of endorsement) in all models of the corresponding media platform predictor across all reports (Curran and Bauer 2010). Hence, results for survey‐level effects are interpreted as deviations from typical. Because within‐person effects are of central interest in the present study, person‐level effects are accounted for in each model but not interpreted (or presented) for parsimony. Models controlled for amount (in minutes) of reported use for the corresponding platform (from 0 to 180), as reported in the baseline survey. The predictors, thus, included survey‐level engagement with media platform (across 10 platforms), the corresponding person‐level aggregate, and minutes of use. Platforms with reported alcohol content of 1% or less are not included in these analyses. Analyses were conducted in Mplus Version 9 (Muthén and Muthén 2017) using maximum likelihood estimation with robust standard errors (MLRs). Missing data were handled using full information maximum likelihood (FIML) estimation, which assumes data are Missing at Random (MAR).

## 3 Results

### 3.1 EMA Response Rates

Average EMA report completion rates were moderate (66% across all scheduled surveys), with the highest response rates at the morning (79%) and nighttime/bedtime (78%) reports; afternoon and evening reports were lower (54% and 52%, respectively). The total number of reports submitted was higher because participants also self‐initiated reports, which could replace a scheduled report for compensation purposes. On average, 88% of participants completed more than one report per day, and more than half (52%) submitted at least three prompted surveys (considered a “complete” day for the purposes of compensation) every day; 63% of participants had “complete” days on 6 of 7 days per week.

**FIGURE 1:**
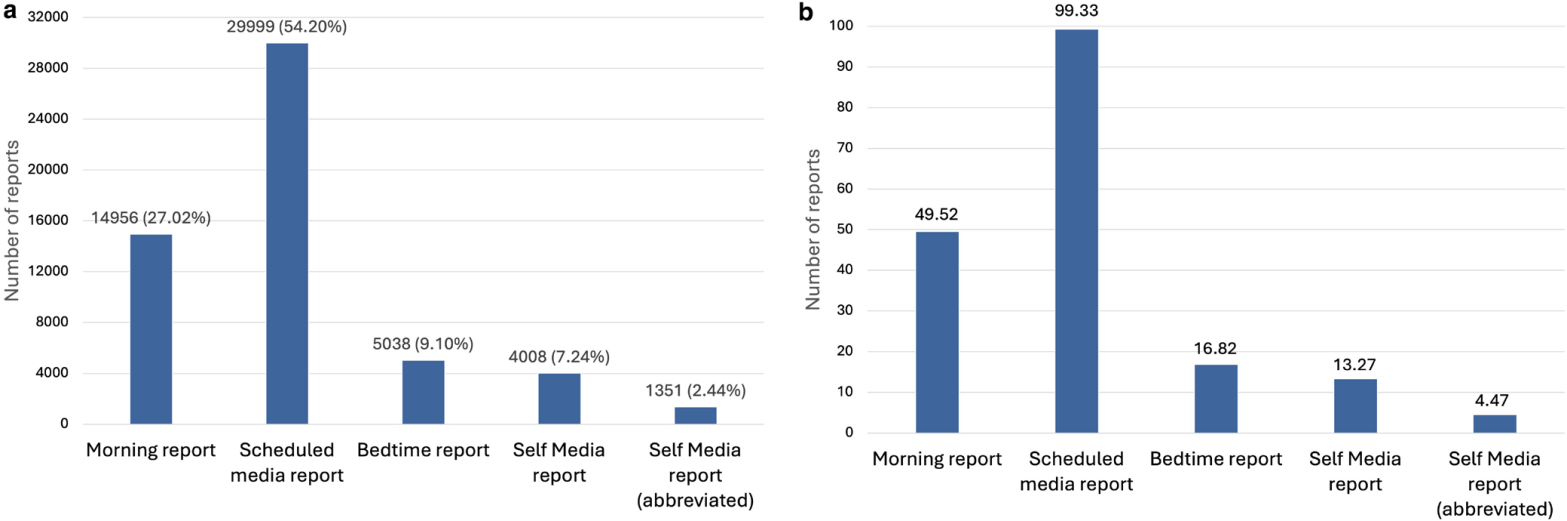
(a) Number of EMA submissions by survey type and by survey. (b) Number of EMA submissions by survey type and by participant.

Across all reports, 55,352 surveys were submitted. Number of submissions by survey type (total and per person) are shown in Figure 1a,b. The majority of reports were from scheduled surveys (27.0% morning reports and 63.3% other scheduled reports), with self‐initiated reports comprising the remainder (9.7%; 5359 of 55,352). On average, participants provided 49.53 morning reports (out of a possible 63) and 116.51 non‐morning scheduled reports (out of a possible 189). They also provided on average another 17.75 self‐initiated media alcohol content reports (ranging from 0 to 205 per person), 4.47 of which were abbreviated, meaning it was not the first alcohol content to be reported within a given time block. Of those 258 participants who self‐initiated a survey, the mean number of reports submitted was 15.53 (SD = 30.37), and of those 219 who completed an abbreviated self‐initiated survey, the mean number of abbreviated reports submitted was 6.17 (SD = 11.17).

### 3.2 General Media Use

Baseline survey data indicated that participants were highly engaged with their personal devices. All but one participant (99.7%) owned their own smartphone (77.6% smartphone, 22.4% Android), and 32% had access to a device through a family member or friend. Among all participants, 87.4% had an unlimited data plan and only 1.3% had no data plan; of those with a limited data plan, only 11.8% had consequences such as paying for additional data or having no access until the following month. The vast majority (93.0%) of participants indicated that the phone remained with them when they slept, with only 4.3% putting the phone in another room and 2.7% giving it to a parent. A full 22.0% did not turn off the phone or turn off notifications while sleeping (e.g., setting the ringer to silent or vibrate). Most (77.2%) reported use of multiple devices simultaneously (e.g., using a tablet and smartphone at the same time) either sometimes or frequently; only 2.6% reported never using two or more devices simultaneously.

Number of hours of media platform use was high, with more than an hour per day for over half of the media platforms, with highest rates for Instagram, TikTok, YouTube, and music (see Table 1). Participants engaged with the vast majority of social media, with 77.2% (Snapchat) to 98.7% (Instagram) of participants interacting in some way. With respect to type of engagement with general media content, liking was the most prevalent reaction, with 91.0% indicating that they sometimes or frequently like content; 61.0% indicated that they sometimes or frequently react to content with an emoji. Following an account (82.1%), saving content (80.8%), and sharing privately (67.5%) on a sometimes or frequent basis also were high. Roughly half of the sample reported sometimes or frequently commenting (56.0%), sharing publicly (52.0%), or clicking a link (53.5%). Blocking a user or account sometimes or frequently was less common but still prevalent (29.4%).

**TABLE 1:** Table of general media platform characteristics, including frequency and magnitude of use and account ownership.

Platform	Spend an hour or more on a typical weekday	Average number minutes/typical weekday	Frequency have an account	Frequency with multiple accounts^a^
Music	91.72%	139	—	—
TikTok	89.70%	98	85.76%	32.89%
Instagram	83.39%	94	99.34%	72.76%
Direct messaging	69.00%	89	—	—
YouTube	63.46%	83	97.33%	22.59%
TV show	56.81%	72	—^b^	—
Movie	43.29%	53	—	—
Video games	41.86%	53	—	—
Snapchat	34.88%	49	82.61%	12.62%
Twitter	26.25%	41	74.09%	18.60%
Discord	25.83%	32	74.33%	15.28%
Pinterest	14.67%	23	70.90%	5.98%
Podcasts	14.57%	18	—	—
Reddit	9.27%	16	55.63%	5.32%
Twitch	6.31%	8	41.67%	1.66%
Facebook	5.67%	13	68.11%	3.99%
Tumblr	3.64%	7	27.48%	3.65%
VSCO	1.99%	6	20.40%	0.33%

*Note:* *N* = 302.

^a^ Of the 242 (80.13%) of participants who reported having more than one account (e.g., fake, spam, alternative, or private) on any social media platform.

^b^ Not relevant for the media platform.

Also shown in Table 1, the vast majority of participants had social media accounts (Instagram 99.3%; YouTube 97.3%; TikTok 85.8%; Snapchat 82.6%); more than half had accounts on Discord (74.3%); Twitter (74.1%); Pinterest (70.9%); Facebook (68.1%); and Reddit (55.6%). Nearly half of the participants engaged in multiplayer video game chatting (46.2%). Many participants reported having multiple accounts on various platforms, particularly on Instagram (72.8%), but also TikTok (32.9%), YouTube (22.6%), and Twitter (18.6%).

### 3.3 In Vivo Exposure to Alcohol‐Related Media

**FIGURE 2:**
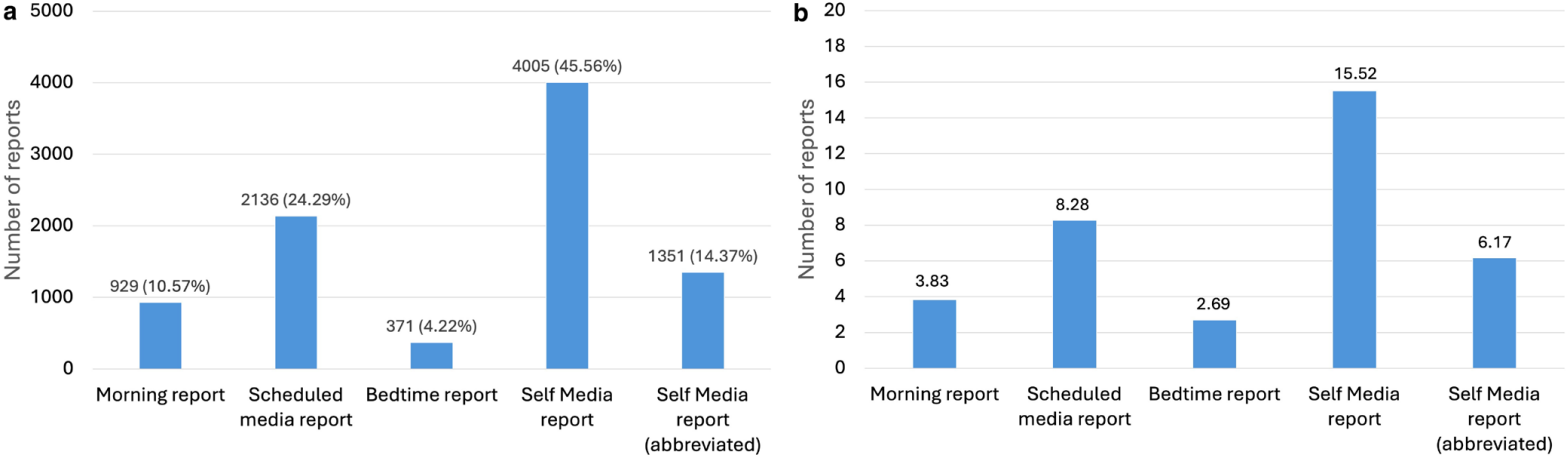
(a) Number of uploads of alcohol content by survey type and by survey. (b) Number of uploads of alcohol content by survey type and by participant.

The total number of reports with alcohol content was 8792, with 300 (of 302; 99.3%) participants providing alcohol media reports (4768 images and 3987 text descriptions; only 37 videos were taken). Figure 2a,b presents the number of media alcohol content submissions by survey type total and by person. The majority of reports were provided in a self‐initiated report (60.9%; 5356 of 8792 reports). One‐quarter (25.2%) of the self‐initiated reports were abbreviated. Alcohol content in scheduled reports was reported across the day, including morning, mid‐day, and nighttime, suggesting that exposures were not necessarily isolated to a certain time of day. Exposure to alcohol content at the person‐level tracked rates at the level of the survey, suggesting that minimal bias occurred due to potential confounding factors, that is, a large number of participants accounting for the majority of reports.

**FIGURE 3:**
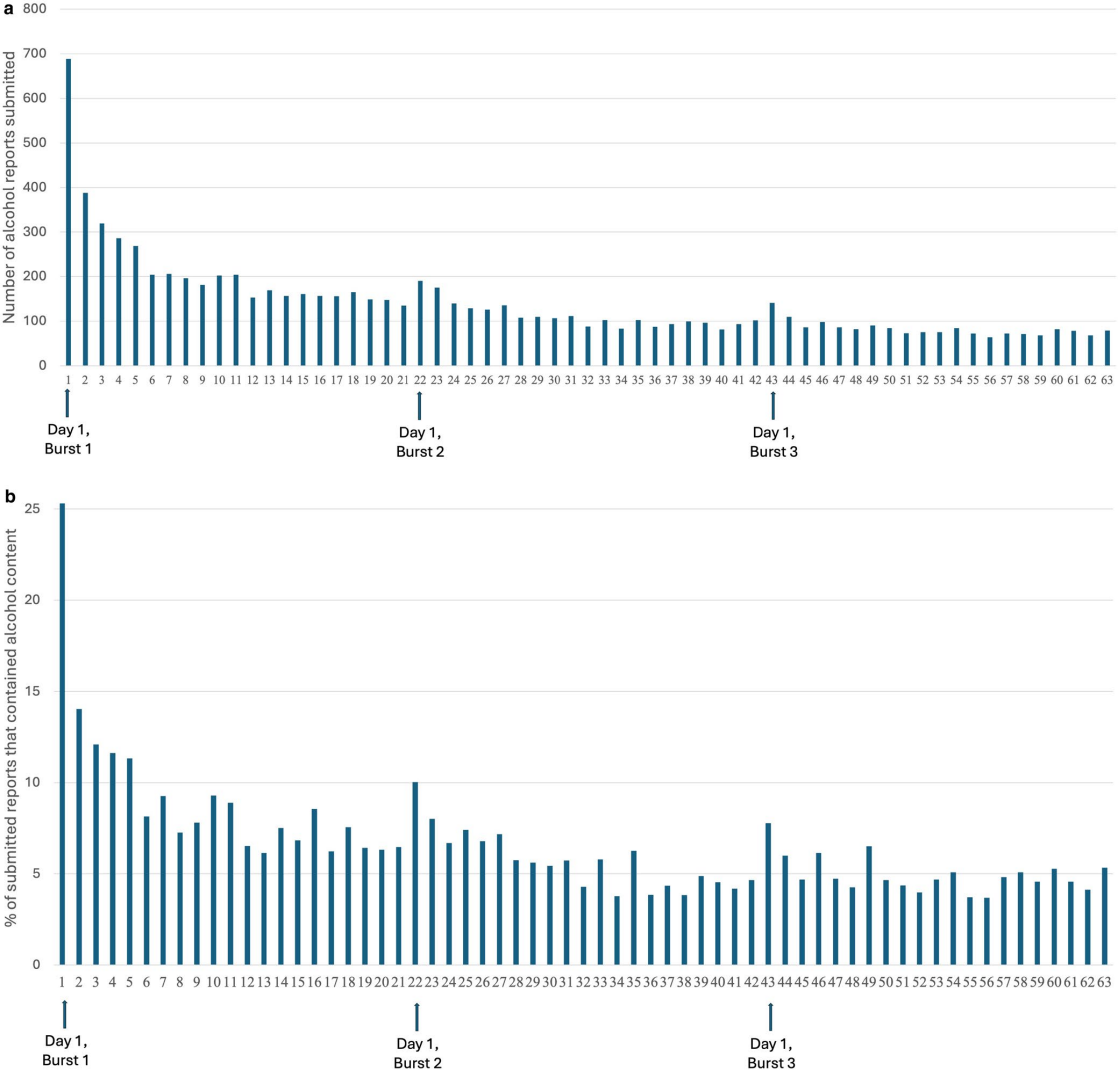
(a) Number of alcohol content reports across the 63 study days. (b) Percent of surveys that included alcohol content across the 63 study days.

Figure 3a,b portrays alcohol content reports across the 63 study days. Unsurprisingly, participants reported the greatest frequency of exposure to alcohol content in the first days in the protocol, followed by an overall decline over the course of the study, but with relative stability within the second and third bursts. To disentangle actual rates of exposure to alcohol content from alcohol report submission, we plotted the percent of scheduled (not self‐initiated) surveys that included alcohol content across study day, given that all self‐initiated surveys had alcohol content by definition. These plots showed a similar pattern, with a relatively gradual decline over the course of the study as well as a decline within burst (although less so for burst three). Reports increased slightly on the first day of each burst.

Table 2 presents rates of exposure to alcohol content by media platform. The platforms on which participants experienced the greatest exposure to alcohol content were Instagram and TikTok, consistent with rates of general media usage, although Instagram was over‐represented in alcohol content, with one‐third of reports from that platform (vs. 18% on TikTok). Although Snapchat, Twitter, and Discord were relatively highly utilized in general (between 25% and 35% reporting spending at least 1 h/day, as reported above), alcohol content was reported primarily on Snapchat among these platforms. Facebook and Twitter contributed relatively low rates of alcohol content, commensurate with relatively low usage in general. Entertainment media platforms were also frequently reported as the source of alcohol content, including music, TV shows, and movies. Although participants reported frequent videogame use, little alcohol content was reported. Also shown in Table 2, the source of alcohol content was an influencer roughly one‐third of the time for social media platforms Instagram, TikTok, YouTube, and Twitter. Nearly half of the YouTube alcohol content, and a large portion of Instagram, Pinterest, and Facebook content, was made, posted, or shared by the alcohol industry.

**TABLE 2:** Exposure to alcohol content across media platforms.

Platform	Media type	Amount of exposure		Content source^a^	
		Number (%) of alcohol content surveys^b^	Mean encounter frequency^c^	Influencer	Alcohol industry
Instagram	Soc media	2855 (32.43%)	9.52	31.07%	29.28%
TikTok	Soc media	1595 (18.13%)	5.32	38.12%	9.40%
TV show	Entertain	1062 (12.09%)	3.54	0.56%	10.73%
Music	Entertain	780 (8.88%)	2.60	0%	0.77%
YouTube	Soc media	682 (7.75%)	2.27	37.54%	53.67%
Snapchat	Soc media	625 (7.17%)	2.08	19.36%	10.56%
Movie	Entertain	329 (3.75%)	1.10	0.91%	4.26%
Pinterest	Soc media	204 (2.32%)	0.68	24.51%	30.39%
Twitter	Soc media	132 (1.50%)	0.44	31.06%	16.67%
Facebook	Soc media	118 (1.34%)	0.39	7.63%	39.83%
Unspecified social media	Soc media	85 (0.97%)	0.28	7.06%	11.76%
Non‐social media/content aggregation site	Other	70 (0.80%)	0.23	4.29%	44.29%
Other	Other	62 (0.70%)	0.21	4.84%	25.81%
BeReal^d^	Soc media	36 (0.41%)	0.12	0%	8.33%
Reddit^e^	Soc media	36 (0.41%)	0.12	8.33%	25.00%
TV/movies‐unspecified	Entertain	22 (0.25%)	0.07	50.00%	59.09%
Video games	Other	15 (0.17%)	0.05	6.67%	46.67%

*Note:* Print media and non‐media write‐in responses are not presented here (*n* = 84).

^a^ % of encounters made, created, or shared.

^b^ *N* = 8792 images or text entries.

^c^ Mean aggregate across 63 days for the 300 participants.

^d^ Item added mid‐survey.

^e^ Category created based on coding of open‐ended text entry responses for “Other” media source. Sources included in “Unspecified social media” include platforms such as Discord, Tumblr, Twitch, and VSCO. Sources included in “Other” include unknown media and podcasts.

### 3.4 Behavioral Engagement With Alcohol Content

In contrast to higher rates of engagement with general social media, engagement with social media alcohol content was the exception rather than the rule. Across all platforms, 25.6% of alcohol reports were liked or received a reaction (e.g., emoji) to the content, but there were few instances of commenting (3.4%) or sharing or posting the content either privately (1.7%) or publicly (0.9%), and only 2.0% of reports were saved or added to a list. Less than 1% of the content was blocked or the user/account deleted (0.8%); clicking on an affiliate link was rare (0.4%), as was following the user/account or a related group (0.3%). Finally, in only 3.3% of the exposures did participants report discussing the alcohol content with someone else. Collapsing across all types, any kind of engagement ranged from 16% (Snapchat) to 45% (TikTok) but hovered around 30% for other social media platforms (32% for Instagram, 28% for YouTube, 36% for Facebook, 25% for BeReal, 30% for Pinterest, 33% for Reddit, 32% for Twitter, and 31% for other/unspecified social media). Although alcohol content was not frequently reported in video games, when it was reported, it was engaged with 20% of the time.

### 3.5 Cognitive Reactions to Alcohol Content

Associations between exposure to alcohol content and our attitude outcomes by media platform, controlling for number of minutes of reported use for the given platform, are shown in Table 3. Platforms that represented < 1% of alcohol content are not shown. The intraclass correlation coefficients (ICCs) indicated that 32% of the variance in attitudes was at the person level (ICC = 0.32), 27% of the variance in norms was at the person level (ICC = 0.27), and 19% of the variance in promote was at the person level (ICC = 0.19). For predictors, ICCs ranged from 0.05 (music) to 0.22 (Snapchat and Instagram).

**TABLE 3:** Associations between exposure to alcohol content and attitudes about the content, by media platform.

Platform	Personal attitudes toward the alcohol content			Perceived peer norms specific to the alcohol content			Belief that the alcohol content promotes alcohol use		
	Est	95% CI	*p*	Est	95% CI	*p*	Est	95% CI	*p*
Instagram	**−0.05**	**−0.01, −0.10**	0.**03**	−0.01	−0.05, 0.03	0.68	**0.13**	**0.08, 0.19**	**< 0.001**
TikTok	0.01	−0.04, 0.06	0.66	0.04	−0.01, 0.08	0.06	**0.05**	**0.001, 0.10**	0.**04**
TV show	0.04	−0.01, 0.09	0.40	−0.04	−0.09, 0.02	0.16	**−0.23**	**−0.29, −0.18**	**< 0.001**
Music	**0.20**	**0.13, 0.27**	**< 0.001**	**0.12**	**0.07, 0.17**	**< 0.001**	−0.004	−0.08, 0.07	0.90
YouTube	−0.02	−0.09, 0.05	0.55	0.04	−0.05, 0.12	0.38	−0.05	−0.15, 0.05	0.32
Snapchat	**−0.27**	**−0.18, −0.36**	**< 0.001**	0.04	−0.03, 0.11	0.25	**0.12**	**0.05, 0.20**	**< 0.001**
Movie	0.07	−0.003, 0.15	0.06	−0.02	−0.08, 0.04	0.54	**−0.21**	**−0.13, −0.29**	**< 0.001**
Pinterest	0.02	−0.17, 0.20	0.85	−0.06	−0.22, 0.11	0.50	**0.26**	**0.18, 0.35**	**< 0.001**
Twitter	0.01	−0.10, 0.11	0.87	−0.08	−0.22, 0.05	0.22	−0.07^a^	−0.23, −0.10	0.41
Facebook	−0.10^a^	−0.21, 0.004	0.06	**−0.13** ^**a**^	**−0.05, −0.21**	0.**003**	0.02	−0.13, 0.17	0.79

*Note:* *N* = 297–299; Number of surveys: 7225–7309. Estimates are unstandardized. Models controlled for number of minutes of reported use for the given platform (ranging from 0 to 180). Platforms with alcohol content of 1% or less are not included. Bold values reach significance at *p* < 0.05 or lower.

^a^ Random slope removed due to convergence difficulties.

Greater exposure to alcohol content in music was significantly associated with both personal attitudes and perceived peer norms favorable toward that content. In contrast, encountering alcohol content on Instagram and Snapchat more than typical was negatively associated with personal attitudes about that content (but not perceived norms). Encountering alcohol content on Facebook was negatively associated with perceived peer norms about that content, but perceived peer norms were not associated with exposure for any of the other media platforms after accounting for typical exposure. Participants believed alcohol content on several social media platforms (Instagram, TikTok, Snapchat, and Pinterest) to promote alcohol use, whereas they believed content on TV shows or movies discouraged alcohol use, controlling for their general tendency to encounter alcohol content on these platforms.

Finally, we explored associations between behavioral engagement in the alcohol content and cognitive outcomes. Any engagement with alcohol content that was more than typical was associated with more positive attitudes toward the content (*est* = 0.36, *p* < 0.001; 95% CI: 0.30, 0.42) and (to a lesser degree) more positive perceived peer norms toward the content (*est* = 0.144, *p* < 0.001; 95% CI: 0.09, 0.18), but negatively associated with the perception that the content promotes alcohol use (*est* = −0.12, *p* < 0.001; 95% CI: −0.07, −0.17)—that is, participants were less likely to engage with content seen to promote alcohol use and more likely to engage with content that discouraged alcohol use.

## 4 Discussion

The present study characterized the nature of day‐to‐day encounters with alcohol content in the media, drawing from rich fine‐grained EMA data collected from a national sample of youth enrolled in high school. This is the first study to characterize real world, in vivo exposure to media alcohol content in detail. Our results showed that adolescents are highly engaged with social media and were frequently exposed to alcohol‐related content in media, on average at least once per week on some of the most saturated platforms. Adolescents also encountered alcohol content on every platform we asked about, with Instagram and TikTok representing the most frequently reported sources of exposure. Immediate reactions to alcohol‐related content on Instagram and Snapchat were personal disapproval about the content, although this did not extend to perceptions of peer (dis)approval of the content. Below, we discuss each of these findings in turn.

Consistent with other research (Graupensperger et al. 2024; Nagata et al. 2025; Pew Research Center 2023; Rideout et al. 2022), participants reported frequent and sustained engagement with media. This included high engagement with their devices and accounts across multiple platforms, frequent use of social media and entertainment media (e.g., music, TV shows, movies), and high rates of account ownership, with many participants reporting multiple accounts on various platforms. Such extensive utilization of media is facilitated by the ease of access on personal mobile devices, which is compounded by simultaneous use of multiple media platforms (with over 97% of our sample indicating doing so) and higher interconnectedness across platforms (e.g., with simultaneous sharing capabilities across TikTok, Instagram, and YouTube). Research increasingly indicates that screen time is a far less informative indicator of risk than the content of the media itself, however (Trager et al. 2023), which may explain why research testing associations between general use of social media and alcohol involvement have yielded inconsistent findings to date. The variability in usage (media platform, amount of use, degree of engagement) in our sample provides further evidence that researchers should not rely on a single index of media “usage.” These findings underscore the importance of this study's detailed characterization of the content users are exposed to in the real world, as well as their reactions to that content.

Our findings also indicated that participants are encountering alcohol content frequently, at different times of the day, and across a wide array of media platforms. The platforms that contributed the most alcohol content are largely the ones that are highly utilized by adolescents in general, and by our study participants. As an example, Instagram was the most highly endorsed social media source of alcohol content, followed by other media platforms popular with adolescents including TikTok, Snapchat, and YouTube, consistent with other work in the field (Steers et al. 2022). Alcohol was rarely encountered on platforms such as Discord, Tumblr, Twitch, BeReal, and VSCO, which may be in part due to platform‐specific restrictions and policies (Berg et al. 2023). Also of note, exposure to alcohol content favoring alcohol use is less likely to be algorithm driven in the latter platforms. This is the first study to report alcohol content on Pinterest but is consistent with observations of waterpipe smoking and e‐cigarette use documented on that platform (Guidry et al. 2016; Lee et al. 2017). Frequent observations of alcohol in TV shows, movies, and in music lyrics were reported, consistent with prior work in the alcohol field as reviewed above. Each of these media platforms facilitates unsupervised, repeated viewing, which compounds the effect. Repeated exposure to media alcohol content over the course of the day, across multiple years, can have powerful impacts on an individual's alcohol‐related cognitions and behaviors. Our findings also showed that nearly a third of all alcohol content reported by participants originated from social media influencers, which is significant given evidence that people often perceive influencers as authentic, relatable, and trustworthy (De Veirman et al. 2017; Leung et al. 2022). It is possible that the association between exposure to alcohol content and drinking behavior is stronger when that content is posted by influencers (Mayrhofer et al. 2020). Finally, extending past research on alcohol‐related social media advertising (Barry et al. 2016; Carah and Brodmerkel 2021), we also found that a significant portion of the alcohol content that users reported was generated by industry brands.

Despite high engagement with social media in general, participants reported minimal engagement with media containing alcohol content and it was almost exclusively in the form of a “like” or emoji‐type of reaction. Our relatively young, alcohol inexperienced sample may have been reluctant to engage in behaviors that seemingly condone heavy drinking and the associated negative consequences (Corcoran et al. 2024; Geusens and Vranken 2021; Merrill et al. 2023). The lower rate of engagement in Snapchat relative to other social media platforms was surprising given its private and ephemeral nature, although it is this very characteristic that may beget posting of more problematic, less socially acceptable drinking patterns (Vranken et al. 2020). Commensurate with their high rates of posting alcohol content (Curtis et al. 2018; Steers et al. 2025), older individuals (young adults and college students in particular), but not adolescents, may actively engage by making comments or sharing content; in contrast, adolescents may find it more enjoyable to passively consume others' posts (Kang and Lou 2022). Future work may derive and measure more passive or non‐quantifiable indicators of engagement such as repeated browsing or skipping content (Kang and Lou 2022) as well as target potential intervention points for selective prevention approaches with youth who are more engaged with alcohol‐related media content.

### 4.1 Exposure to Alcohol Content and Cognitive Outcomes

Upon encountering alcohol content, participants reported their immediate personal attitudes and perceived peer norms (disapproval vs. approval of content, degree of disliking vs. liking content) specific to that content as well as their belief that the content encouraged versus discouraged alcohol use. Alcohol content viewed on Snapchat was strongly associated with disapproving attitudes as was alcohol content posted on Instagram. These two platforms are unique in some ways, for example, disappearing content is one of Snapchat's most defining features and it is possible that it contains portrayals of negative consequences and intoxication that are negatively viewed in our sample of relatively alcohol naïve adolescents. Instagram has traditionally consisted of selected, almost “staged” content versus Snapchat's content that reflects users' activities “in the moment” (Choi and Sung 2018) although Snapchat has become increasingly more filtered and curated. It is possible that teens reported immediate reactions to alcohol content on both of these platforms for different reasons. Alcohol content on Snapchat could be more likely to reflect the negative consequences of alcohol use (e.g., extreme intoxication, embarrassment), whereas alcohol content on Instagram could feel overly “staged” or “fake.” If accurate, interventions could pursue strategies that empower teens to identify and reject overly staged content, consistent with popular media literacy approaches. Both personal attitudes reflecting approval and liking of alcohol content and perceived peer norms were high upon encountering the content on a music app. Yet, on average, participants did not believe that the music content encouraged (or discouraged) alcohol use. It may be that alcohol content in music is mixed in nature, with some elements that portray alcohol in a positive light (partying; sex; humor) but others in a more negative light (violence; use of a vehicle (Primack et al. 2008)). Both TV show and movie content were viewed as discouraging (as opposed to promoting) alcohol use, suggesting perhaps the presence of anti‐drinking content. Only 11% of TV shows and 4% of movies were reported as being made or created by the alcohol industry, though, and younger adolescents may be unable to identify (and report) covertly promoted content (e.g., a character holding branded merchandise) (Patsouras et al. 2024). Future work should explore entertainment media platforms in greater detail with an eye toward identifying anti‐alcohol content. YouTube content was viewed as neither promoting nor discouraging alcohol use, somewhat contradictory with participant report that nearly half of YouTube alcohol content was made, posted, or shared by the alcohol industry. YouTube arguably has the poorest regulation and monitoring (Barry et al. 2016), exacerbated by algorithms recommending other YouTube channels and videos (Kang and Lou 2022), and content analysis of YouTube videos revealed that nearly half contained a brand reference (Primack et al. 2015), similar to our own findings. Understanding why YouTube alcohol content was not associated with cognitive reactions, though, is an area in need of further exploration.

After controlling for the individual's overall exposure to alcohol content on a given platform, few associations were observed between momentary exposure to alcohol content on the platform and the perception that teens their age would approve of or like that content. In general, having the perception that friends approve of drinking is shown to be associated with frequency of posting alcohol‐related content among college students, suggesting a cycle wherein the perceived norms shaped by social media lead to an over‐inflation in norms about posting alcohol content and alcohol use itself (Steers et al. 2019), but these associations may not emerge at the level of momentary exposure. The one exception was alcohol content encountered on Facebook. Facebook tends to be used by an older demographic, and encountering posts of adult drinking such as the Facebook group “Mommy Drinks Wine and Swears” may produce the belief that similar age teens would disapprove of the content. Few adolescents in our sample report using Facebook (< 6%) and thus may not perceive such an association with their own personal attitudes. Despite content posted on Instagram and Snapchat being met with personal disapproval, these cognitions did not extend to perceived peer approval. Prior research has established associations between exposure to alcohol content on these platforms and alcohol use behaviors, and both individual attitudes and perceived peer norms are theorized to shape behavior (RAA: Conner et al. 2017; McEachan et al. 2016); present study findings suggest it may be that personal attitudes but not peer norms link exposure to behavior on these popular and highly utilized media platforms. Future research that specifically tests the proximal links from real‐world media alcohol content to alcohol use is in order. Pinterest was the media platform perceived to most strongly promote (compared to discourage) drinking. These align with what is typical for the platform, for example, Pinterest featuring alcohol favorably (fun cocktail recipes) as opposed to negative consequences (passing out); this distinction can be parsed out with conduct content coding of images, the next step of our line of research. Instagram and Snapchat (and to a little lesser extent, TikTok) content were seen to encourage/promote alcohol use. Instagram is one of the platforms most likely to depict alcohol use as attractive and glamorous (Boyle et al. 2017). Negative portrayals are more characteristic of alcohol content posted on Snapchat (Boyle et al. 2017); perhaps negative alcohol‐related consequences are not necessarily viewed as discouraging of alcohol use (e.g., uninhibited alcohol‐related content such as sexy drunken selfies) (Steers et al. 2022).

### 4.2 Implications for Intervention

This line of research was motivated by the idea that the most opportune time to intervene to reduce the effects of media exposure on drinking is around the time of exposure to alcohol media and/or at the point proximal to where exposure exerts its effects on cognitive mechanisms. Selective prevention approaches may also target youth who are more actively engaging with media alcohol content (e.g., through liking, reacting, or reposting). In recent years, several smartphone‐based interventions that help users respond to ongoing risk factors in the natural environment have shown considerable promise in changing a variety of health behaviors including alcohol use (Carpenter et al. 2020; Haug et al. 2020; Lee et al. 2025). These just‐in‐time (JiT) interventions enable researchers to provide personally tailored intervention at an optimal time for engagement, with the ultimate goal of reducing negative health outcomes such as drinking. Yet, determining which intervention to provide, on what platform, and when requires that researchers know more about the process and timing of media alcohol exposure and its effects on modifiable cognitive/social mechanisms and subsequent drinking. Several findings in this study add key details about where, when, and how adolescents are exposed to alcohol‐related content on social media that could aid in the design of future JiT interventions aimed at reducing exposure to this content or the influence of exposure on behavior. For example, our results showing that adolescents frequently encounter alcohol content on specific platforms like Instagram, TikTok, and YouTube and, less so on other platforms, suggest that interventions might be best focused on these particular venues. Immediate disapproval upon encountering alcohol content on Instagram and Snapchat in particular could be leveraged in preventive interventions. In general, our findings are consistent with the idea that youth interpret alcohol content differently across platform; future work can help us understand how best to introduce platform‐specific media literacy to youth. Moreover, both the large share of reports stemming from influencer content and industry and the power of these advertising strategies in general (Vrontis et al. 2021) suggest that interventions providing additional context to help users process them critically or identify their persuasive intent may be particularly helpful.

### 4.3 Limitations

Although this study had a number of important strengths, several key limitations should also be noted. Our sample was recruited via social media, so their media use rates may be higher than the general adolescent population. While national recruitment is a strength, participants were not nationally representative of the United States, although our rates of past 12‐month drinking rates are commensurate with current population‐based studies (Monitoring the Future; Miech et al. 2025). There may be self‐selection bias, as adolescents who respond to social media recruitment advertisements and who consent to repeated media monitoring may differ systematically from nonparticipants, including being more attentive to and engaged with social media. Given that the inclusion criteria required youth to have consumed alcohol or had a close friend who consumed alcohol, our participants may be more likely to be exposed to alcohol content shared or posted by peers, suggesting possible overestimates of exposure to alcohol content.

Both response rates and reports of alcohol exposures declined over the course of each burst, suggesting that the burden of completing surveys may have led to under‐reporting as the study progressed. Rates of alcohol exposures declined and then stabilized over the course of the study indicating that, although participants began reporting less content (likely to minimize burden), they were still exposed to such content. Interestingly, these trends toward providing fewer reports of alcohol content across the study duration counter the increase that would be observed with alcohol‐related algorithmic targeting, although our study participants self‐reported anecdotally (e.g., to project staff and in open text fields) that they thought they were being targeted. One explanation for the differences in the percentage of alcohol content reports across platforms could be that participants were less likely to report content encountered on particular platforms because it was difficult to capture or recall on that platform (e.g., Snapchat). However, we provided several options for participants to describe the content, including explaining in open text fields what they saw, to avoid penalizing platforms on which specific content is difficult to capture. Additionally, reports were less likely to be submitted in the afternoon and early evening. This pattern suggests that there may be underestimates of alcohol content exposure if some participants are not engaging with their personal devices during these times.

## 5 Conclusions

This study is among the first to provide fine‐grained, real‐world evidence that adolescents are frequently exposed to alcohol‐related content across a wide range of media platforms, particularly Instagram, TikTok, and YouTube, with much of this content on social media originating from influencers and industry sources. Attitudes about alcohol content varied depending on the platform, with more negative attitudes about alcohol‐related content encountered on Instagram and Snapchat. Research that incorporates objective methods of measuring exposure, such as data donation or scraping, is needed to capture a thorough picture of adolescents' media environments. Future research should also explore ways of intervening at or around the time of exposure, either to reduce exposure itself or mitigate its influence on drinking behavior.

## Funding

This study was supported by R01 AA027968 (PI: Kristina M. Jackson) and T32 DA035167 (PI; Linda B. Cottler).

## Conflicts of Interest

The authors declare no conflicts of interest.

## Data Availability

The data that support the findings of this study are openly available in NIMH Data Archive at https://nda.nih.gov/, reference number 10.15154/80ek‐1832.
